# Editorial: Technical Advances in Cryo-Electron Microscopy

**DOI:** 10.3389/fmolb.2019.00072

**Published:** 2019-08-22

**Authors:** Angel Rivera-Calzada, Marta Carroni

**Affiliations:** ^1^Spanish National Cancer Research Center, Madrid, Spain; ^2^Science for Life Laboratory, Stockholm, Sweden

**Keywords:** cryo-electron microscopy (cryo-EM, cryoEM), MicroED, cryo-electron tomography (cryo-ET, cryoET), structural biology, macromolecular complex

Over the last 5 years what has been called the “Resolution Revolution” (Kühlbrandt, 2014) in cryo-electron microscopy (cryo-EM) has opened a new era in structural biology. It is now possible to reveal mechanistic details at near-atomic resolution of new protein machineries, key for understanding how living organisms function (Nogales and Scheres, 2015; Orlov et al., 2017). The level of detail at which structures are now routinely solved using cryo-EM was not possible less than 7 years ago. The wide spread use of cryo-EM has been fuelled by developments in three different areas: transmission electron microscopes optics, software for data analysis and new sensors that combine fast readouts with the ability to directly detect electrons. The revolution is still in progress, and the structural biology community is constantly amazed by the increasing complexity of the macromolecular assemblies being solved at different conformational states, the improvement in resolution for samples below 100 kDa, the *in situ* complexity of the living cells revealed by cryo-electron tomography and the new algorithms for data analysis being constantly published. The pace of advancement is constant and it is important to keep up with it (Danev et al., 2019).

Therefore, with this Frontiers Research Topic entitled “Technical Advances in Cryo-Electron Microscopy” we aim to give an updated and practical overview in different aspects of the cryo-EM field. In this collection, Kim et al. describe a protocol for “Benchmarking cryo-EM single particle analysis workflow” with an overview of the overall process for solving the structure of rabbit muscle aldolase. This protocol is of great interest for both newcomers and more experienced users in charge of EM facilities (Alewijnse et al., 2017; Clare et al., 2017). In the first case it will provide a global view of the process for solving structures using cryo-EM, paying special attention to the current bottlenecks of the process. In the second case it will provide a protocol for standardization and optimization of each stage to guarantee that the best possible data are obtained from the instrumentation.

Sample preparation is still an important limiting factor in achieving high-resolution structures using cryo-EM. Membrane complexes are among the specimens that benefited the most from the new advances in cryo-EM (Cheng, 2018) as they meet the requirements for which cryo-EM is best suited. Membrane complexes are normally difficult to prepare in large quantities and to isolate in their intact form because detergents have to be used. Transmembrane proteins account for less than 3% of the total atomic structures deposited at the Protein Data Bank, while being more than 60% of the current drug targets (Yin and Flynn, 2016). Therefore, the minireview by Sgro and Costa entitled "Cryo-EM grid preparation of membrane protein samples for single particle analysis” will be of great interest for those working in the structural biology field of membrane complexes. In this minireview the authors provide an updated view of novel strategies for solubilization and stabilization of membrane protein samples in order to optimize protein structure determination.

The tendency in the field is that data processing becomes less user-dependent and can be performed in a more automatic way. In reality, different datasets present different limitations, and therefore diverse data processing strategies are of utmost importance to get as much structural information as possible from a given dataset. Serna deals with this topic in her minireview “Hands on methods for high resolution cryo-Electron Microscopy structures of heterogeneous macromolecular complexes.” She discusses different examples especially convenient to deal with one of the main difficulties in data processing which is sample heterogeneity, both conformational and compositional. Besides, she also presents strategies to exploit the presence of symmetry or pseudo-symmetry in the sample in order to achieve as much structural information as possible from our sample.

Most of the development in cryo-EM has been interested in the branch of single particle analysis, where projections coming from multiple copies of the same molecule randomly oriented are taken and combined together to generate a 3D structure. However, electron microscopes are highly versatile and offer also other techniques for 3D structure determination of biological macromolecules (Danev et al., 2019). Tomography followed by subtomogram averaging and diffraction from nano-sized 3D protein crystals, known as microcrystal electron diffraction (microED), are techniques that are gaining ground in the structural biology field.

Cryo-electron tomography allows the study of pleomorphic specimens that are untreatable by single particle projection analysis, such as large complexes in their cellular environment. Subtomogram averaging analysis (STA) is analog to single particle analysis, but volumes, rather than projections are aligned and classified to obtain a medium to high-resolution structure (Wan and Briggs, 2016; Schur, 2019). Our Frontiers Research Topic presents a study by Navarro et al. with a practical walk-through into the usage of the software package Dynamo (Castaño-Díez et al., 2012), a powerful tool for subtomogram averaging, also developed in the same group. Dynamo has been used to publish high-resolution structures by subtomogram averaging (Hutchings et al., 2018) and here the authors present in detail all the steps to bring forward a subtomogram averaging analysis project.

The electron microscope can be used in diffraction as well as in imaging mode and protein as well as small molecule structures can be obtained by the analysis of diffraction spots obtained from micro or nano 3D crystals (de la Cruz et al., 2017; Nannenga and Gonen, 2018). Data are processed using the software developed for X-ray crystallography and microED is particularly attractive for structural biologists with large experience in macromolecular crystallography. With a minireview entitled “The Evolution and the Advantages of MicroED,” Nannenga et al. put in perspective the current approaches for data collection in microED.

In summary with this Frontiers Research Topic we tried to gather contributions from different specialities of life-sciences electron microscopy and we hope that these articles will be useful to get ideas on how to gain structural insights on the biological problem being studied.

## Author Contributions

All authors listed have made a substantial, direct and intellectual contribution to the work, and approved it for publication.

### Conflict of Interest Statement

The authors declare that the research was conducted in the absence of any commercial or financial relationships that could be construed as a potential conflict of interest.
